# Genomic characterization of the Atlantic cod sex-locus

**DOI:** 10.1038/srep31235

**Published:** 2016-08-08

**Authors:** Bastiaan Star, Ole K. Tørresen, Alexander J. Nederbragt, Kjetill S. Jakobsen, Christophe Pampoulie, Sissel Jentoft

**Affiliations:** 1 Centre for Ecological and Evolutionary Synthesis (CEES), Department of Biosciences, University of Oslo, PO Box 1066, Blindern, N-0316 Oslo, Norway; 2 Research Group for Biomedical Informatics, Department of Informatics, University of Oslo, Oslo, Norway; 3 Marine Research Institute, Reykjavík, Iceland; 4 Department of Natural Sciences, University of Agder, Kristiansand, Norway

## Abstract

A variety of sex determination mechanisms can be observed in evolutionary divergent teleosts. Sex determination is genetic in Atlantic cod (*Gadus morhua*), however the genomic location or size of its sex-locus is unknown. Here, we characterize the sex-locus of Atlantic cod using whole genome sequence (WGS) data of 227 wild-caught specimens. Analyzing more than 55 million polymorphic loci, we identify 166 loci that are associated with sex. These loci are located in six distinct regions on five different linkage groups (LG) in the genome. The largest of these regions, an approximately 55 Kb region on LG11, contains the majority of genotypes that segregate closely according to a XX-XY system. Genotypes in this region can be used genetically determine sex, whereas those in the other regions are inconsistently sex-linked. The identified region on LG11 and its surrounding genes have no clear sequence homology with genes or regulatory elements associated with sex-determination or differentiation in other species. The functionality of this sex-locus therefore remains unknown. The WGS strategy used here proved adequate for detecting the small regions associated with sex in this species. Our results highlight the evolutionary flexibility in genomic architecture underlying teleost sex-determination and allow practical applications to genetically sex Atlantic cod.

Teleosts are characterized by a remarkable diversity of independently evolved sex-determining mechanisms that range from those using environmental cues to those under strict genetic control^1^,^2^,^3^,^4^. Different genes control sexual fate in a variety of teleosts and a variety master sex determining (SD) genes have been described, *dmY*, *gsdfY* and *sox3Y* in the medaka genus^5^,^6^,^7^,^8^, *amhr2* in fugu^9^, *amhy* in Patagonian pejerry^10^ and Nile Tilapia^11^, *dmrt1* in half-smooth tongue sole^12^, *gdf6Y* in Killifish^13^ and *sdY* in rainbow trout^14^. Of these examples, only *sdY* represents a case whereby a gene without previously known functionality during sexual development has been recruited as a SD gene, and could be an example of *de novo* SD evolution^4^,^14^. The potentially rapid evolution of SD mechanisms^15^ and their striking diversity, even in closely related lineages like those of the medaka genus^16^ and the stickleback family^17^,^18^,^19^ hinders the identification and characterization of such mechanisms in divergent teleost lineages.

Here, we characterize the sex-locus in Atlantic cod (*Gadus morhua*), an economically and culturally important marine resource in the North Atlantic region. In this species, divergent and sex-dimorphic expression between females and males has been observed for several genes, including copies of *dmrt*^20^, *sox9* and *cyp19a1*^21^ and several estrogen receptors (ER)^22^. Nevertheless, these genes regulate the downstream process of sex differentiation, and there is no direct evidence for their role in sex-determination. Therefore the identity of the sex-locus in Atlantic cod has remained unknown.

Atlantic cod exhibits variable levels of sexual dimorphism, with females becoming longer and heavier than males^23^. Sex further alters the propensity to mature early in aquaculture, with males maturing earlier than females, leading to reduced male somatic growth, resulting in considerable economical loss^24^. Several observations provide evidence for a largely genetic rather than environmental sex determination in Atlantic cod. First, equal male to female ratios are found in wild Atlantic cod specimens below 60 cm^25^, regardless of the highly skewed sex ratios that can be observed during spawning^26^. Equal sex ratios are also obtained in controlled experimental crosses^27^. Finally, the generation of all-female populations by gynogenesis or breeding with masculinized females provides not only evidence for genetic sex-determination, but also for male-heterogametic sex-determination, i.e. a XX-XY system^27^,^28^.

We analyzed whole genome sequence (WGS) data from 227 Atlantic cod specimens in order to find the putative location of the sex-locus in this species. First, the analysis of a stringently filtered SNP dataset containing genotypes from 48 individuals identifies a single genomic region on linkage group (LG) 11 (sensu Hubert *et al*.^29^) that contains the only genotypes that segregate according to a male-heterogametic sex-determination system in this reduced dataset. Subsequently, we confirmed this regions’ association with gender by determining genetic sex in 179 unrelated individuals of known sex. Through analysis of an unfiltered variant dataset of all 227 individuals, we identified five additional genomic regions on alternate linkage groups that can be associated with sex, although this association is inconsistent. The reference genome has been based on data from a male specimen. To exclude large-scale assembly errors as the most likely explanation for these sex-linkage patterns, PacBio read data obtained from a female individual was used to independently confirm assembly accuracy. The identified sex-associated region on LG11 is less than 55 Kb and lacks clear sequence homology compared to genes or regulatory regions previously identified as sex-locus. Moreover, it is not directly associated with genes known to be involved in sex differentiation. The functionality of this region remains therefore obscure.

## Results

We obtained average Illumina sequence coverage of 10.9 X per individual (Supplementary Table 1), retaining 1,573,340 SNPs after filtering and 55,160,622 variable sites without filtering. Out of these variants, we selected those polymorphisms with a maximum of two heterozygote genotypes in the homogametic sex, and a maximum of two homozygote genotypes in the heterogametic sex in a subset of 21 males and 27 females. Following our criteria, three and thirteen loci show sex-linked segregation (homozygous females and heterozygous males) using the filtered or unfiltered dataset, respectively (Table 1). Thus, filtering using recommended practices substantially reduces the number of loci that are identified, including several insertion-deletion polymorphisms. All of the identified loci in the subset of individuals are located within a single, relatively small genomic region on linkage group (LG) 11. In contrast, the reverse selection criteria (heterozygous females and homozygous males) do not yield a single polymorphism.

We calculated individual values of the inbreeding coefficient (F) based on the 13 identified sex-linked polymorphisms. Positive F values indicate largely homozygous and negative F values heterozygous genotypes. By classifying individuals based on these F values, we confirm female or male phenotypic sex in a total of 179 unrelated specimens, misclassifying a single individual. Moreover, the 13 polymorphisms display distinct homo- or heterozygous segregation depending on sex (i.e., the majority of individuals is either fully homozygous or heterozygous, Supplementary Table 1). The single misclassified specimen has heterozygous genotypes for all 13 loci and is therefore clearly a genetic male. The probability of obtaining 13 heterozygous loci in a female given random genotype error or recombination is vanishingly small. We therefore postulate that human error (most likely while recording sex) is responsible for this single misclassification.

**Figure 1:**
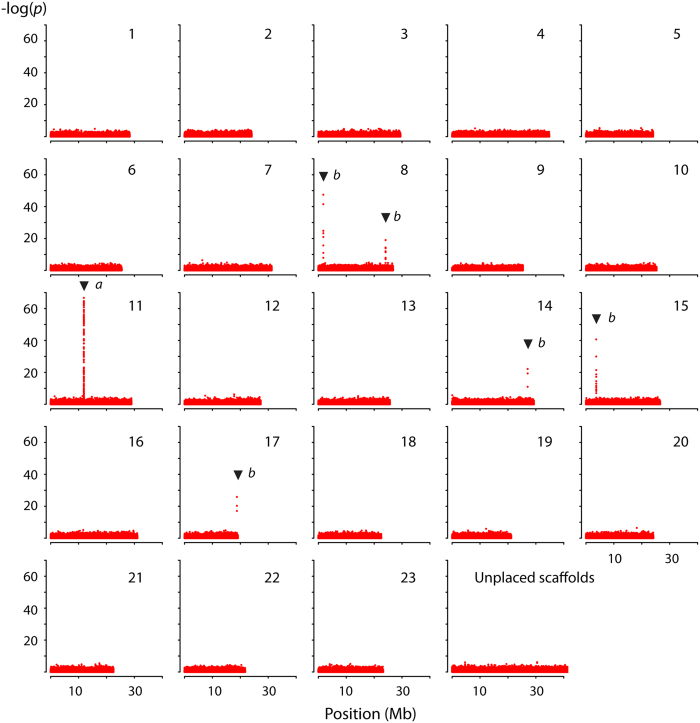
Sex linked segregation of genotypes in Atlantic cod. Over 55 million polymorphisms are compared in 110 males and 116 females of Atlantic cod using Fishers’ exact test. Six distinct regions are identified with a significant increase in *p*-values, i.e. above 6SD of the mean. Highest numbers of genotypes with most extreme *p*-values are found on LG11 (a), whereas reduced numbers with lower *p*-values are found on LG08, LG14, LG15 and LG17 (b). *P*-values were calculated using PLINK (v.1.90p) and −log transformed. Unplaced scaffolds have been concatenated for visualization.

By further investigating sex-linked genotypic segregation in more than 55 million polymorphisms using the unfiltered dataset for 110 males and 116 females (excluding the misclassified individual), we identify six distinct regions with elevated *p*-values (i.e., six times above the standard deviation (SD) of the mean transformed *p*-value) on five different LGs using Fishers’ exact test (Fig. 1). LG11 contains the largest region (55 Kb) and the highest number of sex-linked loci (*n* = 127) with most extreme *p*-values, whereas the other LGs contain relatively small regions and lower number of less extreme *p*-values (Table 2). Among all sex-linked loci, those with transformed *p*-values above 50 (*n* = 36) show a near exclusive XX-XY segregation of genotypes, although genotypes with inconsistent sex-linkage do occur in that range (Supplementary Table 2). Loci with *p*-values below 50 exhibit a higher proportion of genotypes that are inconsistently linked to sex-specific segregation (Supplementary Table 2).

**Figure 2:**
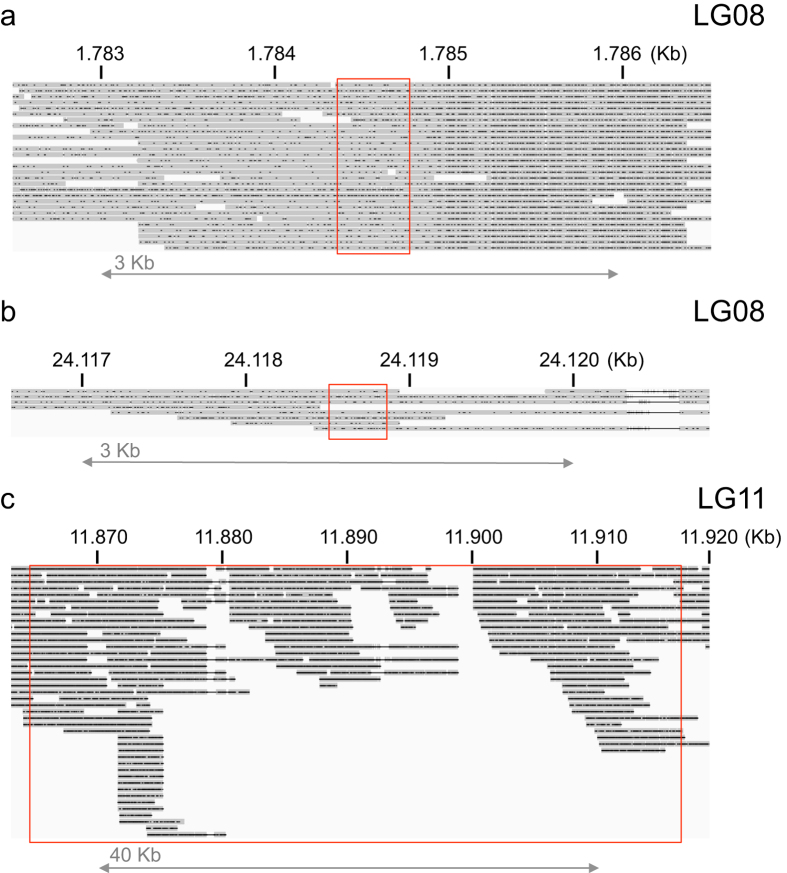
PacBio read alignments of a female Atlantic cod specimen. Long read alignments (grey) towards the male gadMor2 reference genome overlap those regions containing sex-associated genotypes (red box) on LG08 (**a,b**) and LG11 (**c**). Note the different genomic scale (in Kb) for each of the sub-panels. Small indels (black dots) within the alignments are a typical feature of PacBio read data. Read alignments are visualized using the Integrative Genomics Viewer.

It is possible that assembly errors are causal for the observed pattern of multiple sex-associated regions. We independently assessed the assembly accuracy of these regions by aligning PacBio reads from a female specimen towards the gadMor2 genome^30^. We obtain long-range coverage for all sex-linked regions (Fig. 2, Supplementary Figures 1–3) and do not observe alignment failures that indicate large-scale assembly errors at the edges of these regions. Nevertheless, within sex-associated region on LG11, no female PacBio alignments can be observed crossing an approximately 1 Kb section around position 11,900,000 bp (Fig. 2C), which can be indicative of assembly error or the presence of larger, female specific genomic rearrangements that cause long-read alignments to fail. Should the latter be the case, we hypothesized that should observe PacBio read alignments crossing this region if we aligned their read ends only, essentially like a paired-end read. By aligning such artificially created “paired-end” PacBio reads, we indeed observe 19 instances of female PacBio reads crossing the region on LG11 (Supplementary Figure 4), further supporting the current orientation of the genome assembly. Overall, we conclude that is unlikely that large-scale assembly errors explain the observed associations with sex over multiple linkage groups.

**Figure 3:**
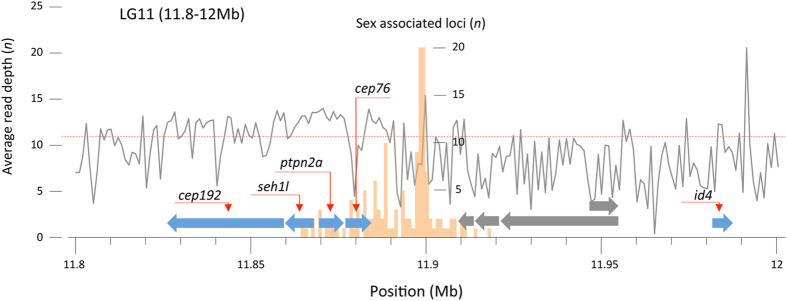
Annotation surrounding the sex-associated region of Atlantic cod on linkage group 11. Nine gene models (the arrow shows transcriptional direction) have been annotated within a 200 Kb window around the loci with sex-linked genotypic segregation. The histogram (orange) shows the number of sex associated loci (with *p*-values more than 6 SD from the mean) in windows of 1 Kb. Gene models with cDNA evidence from Atlantic cod (blue) have been annotated with gene names and are in conserved synteny (order and transcriptional direction) with those in three-spined stickleback and spotted gar. Three genes (*ptpn2a, cep76 and id4*) are in conserved synteny with medaka. Gene models without cDNA evidence (grey) do not have obvious homology with known genes. Average read depth (grey line) is calculated in 1 Kb windows and can be compared to the genome-wide average coverage (red dashed line).

We investigated the locations surrounding the sex-linked regions for presence of genomic features that can provide insight in their function. We do not observe a direct association of the identified regions with any of the candidate genes known to be involved in sex-determination of other fish, or with genes known to be involved in sex-differentiation in Atlantic cod. Instead, the most likely alignments of those candidate genes are found on other linkage groups or are located at least 4 MB away (e.g. *dmrt4* on LG17, Table 3). The sex-linked regions on LG14, 15 and 17 occur completely or partly in dispersed repeats (LTR/Copia, data not shown), those on LG8a are associated with an unknown protein model, whereas those on LG8b are not associated with any automated annotation (data not shown). Given that these regions are relatively small and inconsistently sex-linked, we further focus on a 200 Kb region around the sex-linked loci of LG11. Within this region, we find evidence for nine protein coding gene models however; the highest number of sex-linked genotypes is not associated with any of these (Fig. 3). Specifically, the majority of genotypes are located downstream of an annotated gene (*cep76*) and an unnamed gene model (without evidence for a particular protein homology).

Of the nine gene models, four have no identified orthologs in other species and have no transcription evidence based on Atlantic cod cDNA data. The five genes with transcriptional evidence (annotated *cep192, seh1l, ptpn2a, cep76* and *id4*) based on Atlantic cod cDNA data^30^, also have high sequence homology towards genes in other species. This region is similarly annotated in the first Atlantic cod genome assembly on GeneScaffold_1523^31^, although that assembly contains substantially more sequence gaps –in particular around the location of the sex-locus. Within the 200 Kb region, the five genes with high sequence homology towards known genes show conserved synteny based on order and transcriptional direction with several other fish species, for instance stickleback (*chrXX: 1,45-1,6 *Mb) and spotted gar (*LG11: 32,28-32,56 *Mb). Conserved synteny for three of these (*ptpn2a, cep76* and *id4*) is observed in medaka (*chr16: 1.4-1.7 *Mb).

## Discussion

Here, we identify several genomic regions in Atlantic cod that contain loci with evidence for sex-linked genotypic segregation. Nonetheless, the region on LG 11 shows most evidence for being involved in sex determination. First, this region is an order of magnitude larger than the others, and contains the highest number of sex-linked loci. Second, those loci in this region are the most strongly segregating according to the male-heterogametic sex-determination system that is expected from earlier experimental crosses^28^. Our data yield no support for a female heterogametic system (i.e., ZZ-ZW system). Finally, based on 13 loci within the region on LG11 that have strict male-heterogametic sex segregation, we can confirm phenotypic sex with genotypic sex in 178 unrelated individuals obtained from geographically separated populations, whereas sex is imperfectly linked in the other genomic regions. Taken together, we conclude that the most likely location for the sex-locus in Atlantic cod is located on LG11 within this 55 Kb window.

The relatively small genomic footprint of the sex-associated regions observed in Atlantic cod limits the type of sequencing strategy that can be used for their detection. Specifically, reduced representation sequencing of genomic DNA (e.g. ddRAD-seq, double digest restriction site – associated DNA sequencing) has been proposed as economically efficient approach for genotyping non-model species^32^. Using such approach in rockfish, up to 33 sex-specific loci were obtained out of a total of 74,965 loci^33^. We here identified 166 sex-associated loci out of a total of 55,160,622 variable sites at a rate of 1 in 332,292 loci. Thus, with our observed discovery rate, it is doubtful that a reduced representation sequencing strategy would have yielded sufficient information to characterize the sex-linked regions or deliver reliable sex-linked genotypes. Should similarly sized sex-loci as observed in Atlantic cod be more widespread among teleosts, reduced representation sequencing approaches will likely be of limited use in their detailed characterization.

Automated annotation of the 200 Kb region surrounding the 55 Kb window does not provide strong evidence about the functionality of the sex-locus. We find no sequence homology with candidate SD genes from other teleosts^6^,^7^,^8^,^9^,^10^,^14^ that are all located on different linkage groups. Similarly, no genes reportedly involved in sexual differentiation in Atlantic cod^20^,^21^,^22^ are located on the same linkage group. No *ab initio* gene model is directly associated with the region that is most strongly associated with sex-specific segregation (Fig. 3). This region’s lack of clear sequence homology and lack of direct association with known candidate genes leaves its functionality unknown. Moreover, based on conserved synteny of five genes (*cep192, seh1l, ptpn2a, cep76* and *id4*) surrounding the Atlantic cod sex-associated region in teleosts like stickleback, spotted gar and medaka, this region is likely of ancient evolutionary origin. In these other species however, no role for sex-determination has been suggested for this location. Therefore, the sex-locus in Atlantic cod appears to be a derived, non-homologous feature. Two scenarios may explain the observed lack of sequence homology; 1) Atlantic cod has recruited a novel type of sex determining locus (with unknown function). 2) Atlantic cod has retained a known locus, yet this subsequently evolved and diverged to such extent that sequence homology analyses fail to detect this locus, whilst maintaining its original functionality. In both scenarios, this observation records extensive evolutionary divergence in the genomic architecture underlying sex-determination. Should the sex-determining locus in Atlantic cod be newly recruited, this adds to a growing body of literature suggesting greater genetic plasticity in the sex-differentiating cascade^4^ than previously anticipated^34^.

We note that the sex-associated region is located ~80 kb upstream from *id4*, which encodes a transcription factor of the inhibitor of DNA binding (ID) protein family. One of the related pathways of *id4* is the TGF-β signaling pathway^35^. Variation in genes of the TGF-β signaling pathway plays a role in the sex determination of a variety of fish. For instance gene duplicates of Anti-Mullerian Hormone (*amh*) determine sex in Patagonian pejerry^10^ and Nile Tilapia^11^, a missense SNP in the Anti-Mullerian Hormone receptor II (*amhr2*) determines sex in fugu^9^ and male specific variation in a TGF-β growth factor (*gdf6Y*) determines sex in killifish^13^. It may be that male-specific regulation of *id4* initiates a downstream cascade through the TGF-β signaling pathway, which eventually results in gender determination in Atlantic cod.

The identification of the Atlantic cod sex-locus has several practical applications. For instance, phenotypic sex is difficult or impossible to obtain if specimens are not large enough for a visual assessment of gonads (i.e. early larval stages) or if fish are in non-spawning condition and preferentially kept alive. Our findings provide the means to retroactively assign genetic sex. Moreover, our finding allows the assignment of the sex phenotype for specimens for which this is impossible to obtain by other means, such as archeological bone material^36^ or historical specimens^37^,^38^ of unknown sex. Finally, this finding eases the identification of masculinized females for the generation of all-female populations^28^, providing opportunity for a more profitable aquaculture.

## Methods

### Ethics statement

We always strive to limit the effect of our sampling needs on populations and individuals. Therefore, we collaborate with other sources, like commercial fisheries or aquaculture farms, from which samples can be obtained as a byproduct of conventional business practice. This way, no animals need to be sacrificed to serve our scientific purpose. Samples were taken *post-mortem* and no scientific experiments have been performed on live animals. All specimens were part of larger hauls, caught by commercial vessels, were euthanized by local fishermen and were intended for human consumption. Sampling in this manner does not fall under any specific legislation in Norway or Iceland. These methods are in accordance with the guidelines set by the ‘Norwegian consensus platform for replacement, reduction and refinement of animal experiments’ (www.norecopa.no).

### Sampling and DNA extraction

Atlantic cod specimens (*n* = 227) were obtained from several locations and sample dates around Norway and Iceland (Supplementary Table 3). Sex was determined through visual assessment of male (n = 112) or female (n = 115) gonads for each individual. DNA was extracted from fin clips, muscle or spleen using DNeasy Blood & Tissue kit (Qiagen), and sheared to an approximate insert size of 350 bp. Over 2 μg of DNA per sample was used to create Illumina compatible sequencing libraries using the TruSeq DNA PCR-Free LT Library Preparation Kit (Illumina). Libraries were individually barcoded, pooled and sequenced together on a HiSeq 2000. After demultiplexing with Illumina RTA (1.18.61.0) & CASAVA (1.8.4), sequencing reads were aligned using the mem algorithm of BWA v.0.7.5a-r405^39^ to the gadMor2 genome assembly^30^ available from the European Nucleotide Archive (ENA) LN845748-LN845770. Polymorphic variants were jointly called for all 227 individuals using GATK v. 3.3.0^40^ according to GATK Best Practices recommendation^41^,^42^. For the filtered dataset, we selected sites with a minimum quality (FS > 60.0 || MQRankSum < −12.5 || ReadPosRankSum < −8.0 || QD<2.0 || MQ<40), allowing no SNPs within 10 base pair (bp) of an indel with BCFTOOLS v. 1.2^43^. After this, we removed all indels and SNPs with more than two alleles, an average read depth higher than 30, a minimum allele frequency (MAF) below 0.05 and SNPs with more than 10% missing data per population using VCFTOOLS v. 0.1.14^44^. Both datasets –filtered and unfiltered– were used for subsequent analyses.

### Identification of putative sex-locus

To identify the location of the putative sex-locus, we use two approaches. First, we analyzed 48 individuals sampled in Lofoten, Norway 2011 (♂ = 21, ♀ = 28, Supplementary Table 1) to identify SNPs and other biallelic polymorphisms with a strict sex-specific segregation (i.e. those either exclusively homozygous or heterozygous depending on sex) using VCFTOOLS v. 0.1.14^44^. Specifically, we select all polymorphisms with a maximum of two heterozygote genotypes in the homogametic sex, and a maximum of two homozygote genotypes in the heterogametic sex (heterozygote genotypes have a tendency to be lost from low-coverage genome data^45^), allowing for 10% missing data. Those polymorphisms that agree with these criteria are subsequently used to determine genetic sex in the other 179 specimens by calculating the inbreeding coefficient, F, for each individual using a method of moments using the –het algorithm in VCFTOOLS v. 0.1.14^44^. Individuals with positive F values (e.g. more homozygous than expected by chance) are classified female, whereas those with negative F values are classified male. We investigated the putative sex determining genomic region using the unfiltered whole genome variants dataset of 110 males and 116 females –excluding a single misclassified individual. We identify sex linked segregation of genotypes by calculating exact *p*-values using Fishers’ exact test with the options –fisher –model in PLINK v. 1.90p^46^. This algorithm is general test of association in the 2-by-3 table of genotypes rather than the basic allelic test (which compares frequencies of alleles in cases versus controls). For visualization, *p*-values were –log transformed.

### Alignment of female PacBio reads

Long-range PacBio reads (P6-C4 chemistry) of a female Atlantic cod specimen were aligned to the gadMor2 assembly^30^ using BLASR v.3.0.1^47^ with the following options: -bestn 2 -clipping subread -affineAlign -noSplitSubreads -nCandidates 20 -minPctIdentity 40 -sdpTupleSize 6. Based on the long-range PacBio data of the female specimen, we created artificial paired-end reads with a length of 300 bp for the forward and reverse reads, using prinseq-lite v.0.20.4^48^. These paired-end reads were subsequently aligned using the mem algorithm of BWA v.0.7.5a-r405^39^. Aligned full-length PacBio reads and artificial paired-end PacBio reads were visualized using the Integrative Genomics Viewer^49^.

### Location of candidate SD or sex differentiating genes

We identify the location of previously known SD genes or those implicated in sexual differentiation using the following approach. Candidate genes (*akap11, amh, amhy, amhr2, ar, cyp19a, cyp19b, dmrt(2a,3,4,5), dmy, esr1, esr2a, esr2b, foxl2, gsdf, sdY, sox3, sox9a, sox9b* and *vasa)* were aligned to the Atlantic cod genome using exonerate 2.2.0^50^ with the option –model coding2genome. Alignments with the highest score were selected as the most likely genomic location in the Atlantic cod genome using the option –bestn.

### Protein annotation of sex-determining region

Protein annotation of the Atlantic cod genome (gadMor2) is described in detail elsewhere^30^. In short, we used MAKER2, v. 2.31^51^,^52^ to combine the consolidated evidence from *ab initio* gene finders and physical evidence (e.g. protein and RNA sequence alignments) in to a set of quality gene models. Evidence from RNA sequence alignments was obtained using a combined RNA-sequence dataset from various sources^31^,^53^,^54^ including a set of PacBio reads^55^. Gene models were aligned using BLAST^56^ against the SwissProt/UniProtKG release 2015_12 to obtain putative gene names.

### Data availability

The gadMor2 genome assembly is available from the European Nucleotide Archive (ENA) LN845748-LN845770. The annotation of the Atlantic cod genome is available from https://t.co/mdiVe52v4d. All individual read data (including the female PacBio data) associated with linkage groups containing sex-linked genotypes are available from ENA with study accession number PRJEB14672.

## Additional Information

**How to cite this article**: Star, B. *et al*. Genomic characterization of the Atlantic cod sex-locus. *Sci. Rep*. **6**, 31235; doi: 10.1038/srep31235 (2016).

## Supplementary Material

Supplementary Information

## Figures and Tables

**Table 1:** Polymorphisms with sex-linked genotypic segregation in 48 Atlantic cod specimens (21 ♂ and 27 ♀).

LG	Position (bp)	Allele		Genotype (Ref/Het/Alt^1^)	
		Reference	Alternative	Females	Males
11	11885753^2^	G	T	0/2/24	1/20/0
11	11886873^2^	T	A	0/0/27	1/20/0
11	11888434	T	C	27/0/0	0/21/0
11	11893118^2^	T	A	27/0/0	1/18/1
11	11897471	G	GTGT	0/0/27	0/21/0
11	11897513	C	T	0/0/27	0/20/1
11	11897519	A	T	0/0/27	0/21/0
11	11897566	AATCC	A	0/1/25	1/19/0
11	11899188	A	G	0/0/27	0/20/1
11	11899196	G	T	0/0/27	0/21/0
11	11899391	C	CT	0/0/27	0/21/0
11	11899539	G	T	0/2/25	0/20/0
11	11899548	TA	T	0/2/25	0/20/0

Limited numbers of polymorphisms (3 out of 1,573,340 in the filtered, 13 out of 55,160,622 in the unfiltered dataset) are identified, which have highly homozygous genotypes for females and heterozygous genotypes for males. All polymorphisms are co-located within a 15 Kb region on linkage group (LG) 11.

^1^Ref = homozygous reference, Het = heterozygous, Alt = homozygous alternative.

^2^Identified in the filtered SNP dataset.

**Table 2:** Linkage groups and regions that contain loci with sex-linked segregation in 226 Atlantic cod specimens.

LG	Start (bp)	Stop (bp)	Size (bp)	Sex-linked (*n*)	Total (*n*)	Max-*p*
08a	1784400	1784813	413	11	23	47
08b	24118524	24118864	340	8	14	19
11	11864114	11918378	54264	127	472	67
14	27062221	27062388	167	3	8	22
15	3678047	3679605	1558	14	64	41
17	18769676	18769822	146	3	6	26

Start and Stop refer to the location of the first and last locus with a high (greater than six standard deviation from the mean) Fisher’s exact test *p*-value in a particular region. Size is calculated as the difference between Start and Stop. For each region, the number of sex-linked loci, total number of loci (including those that are not linked to sex, using a MAF of 0.05) and the maximum *p*-value (Max-*p*) observed in that region are given.

**Table 3:** Names and genbank ID of sex-determining genes from various teleosts and genes known to be involved in sex-differentiation in Atlantic cod.

Gene name	Genbank ID	Species	LG	Start	Stop	Alignment score
akap11	XM_011473624.1	Oryzias latipes	20	10120287	10118045	1859
amh	JN802292.1	Gadus morhua	12	24019786	24018572	1610
amhy	HM153803.1	Odontesthes hatcheri	12	24019643	24018570	473
amhr2	NM_001280009.1	Takifugu rubripes	13	9249009	9250493	574
ar	FJ268742.1	Gadus morhua	10	19432117	19442119	3743
cyp19a	DQ402370.1	Gadus morhua	14	16165860	16169755	2851
cyp19b	JN802291.1	Gadus morhua	9	2257039	2260649	2648
dmrt	AJ506094.1	Gadus morhua	6	19949821	19940091	855
dmrt2a	JN802284	Gadus morhua	6	19911488	19908495	1650
dmrt3	JN802285	Gadus morhua	6	19922948	19919278	2020
dmrt4	JN802286	Gadus morhua	17	14093750	14092035	2546
dmrt5	JN802287	Gadus morhua	12	20988494	20990196	2537
dmy	NM_001104680.1	Oryzias latipes	6	19948867	19940028	382
esr1	JX178935.1	Gadus morhua	21	18435483	18415167	2611
esr2a	JX178936.1	Gadus morhua	21	8193571	8213026	3711
esr2b	JK993476.1	Gadus morhua	5	20297385	20297568	334
foxl2	NM_001104888.1	Oryzias latipes	1	27838167	27837834	470
gsdf	KC204828.1	Gadus morhua	4	25085824	25083577	1040
sdY	NM_001281416.1	Oncorhynchus mykiss	7	1898620	1898410	136
sox3	AB775143.1	Oryzias dancena	10	17937590	17936636	1482
sox9a	JN802288.1	Gadus morhua	18	7659675	7661680	1722
sox9b	JN802289.1	Gadus morhua	2	17478629	17480857	2714
vasa	HM451456.1	Gadus morhua	7	3034506	3016733	3812

Location (in base pair) on the various linkage groups (LD) was determined by aligning the protein coding sequence to the genome assembly using exonerate 2.2.0 50 with the option –model coding2genome. Alignments with the highest score (–bestn) were selected as the most likely genomic location.
