# Supplementary material for: Genomic characterization of the Atlantic cod sex-locus
Source: Sci Rep. 2016 Aug 8;6:31235. doi: 10.1038/srep31235 (PMC4976360; doi:10.1038/srep31235)
Supplement: Supplementary Information [file srep31235-s1.pdf]

Supplementary Information

Genomic characterization of the Atlantic cod sex-locus

Bastiaan Star[1], Ole K. Tørresen[1], Alexander J. Nederbragt[1,2], Kjetill S. Jakobsen[1], Christophe Pampoulie[3], Sissel Jentoft[1,4]

[1] Centre for Ecological and Evolutionary Synthesis (CEES), Department of Biosciences, University of Oslo, PO Box 1066, Blindern, N-0316 Oslo, Norway.

[2] Research Group for Biomedical Informatics, Department of Informatics, University of Oslo, Oslo, Norway

[3] Marine Research Institute, Reykjavík, Iceland

[4] Department of Natural Sciences, University of Agder, Kristiansand, Norway

Corresponding author

Bastiaan Star (bastiaan.star@ibv.uio.no)

Keywords:

Sex-determination; sex-locus, sex-differentiation, Atlantic cod

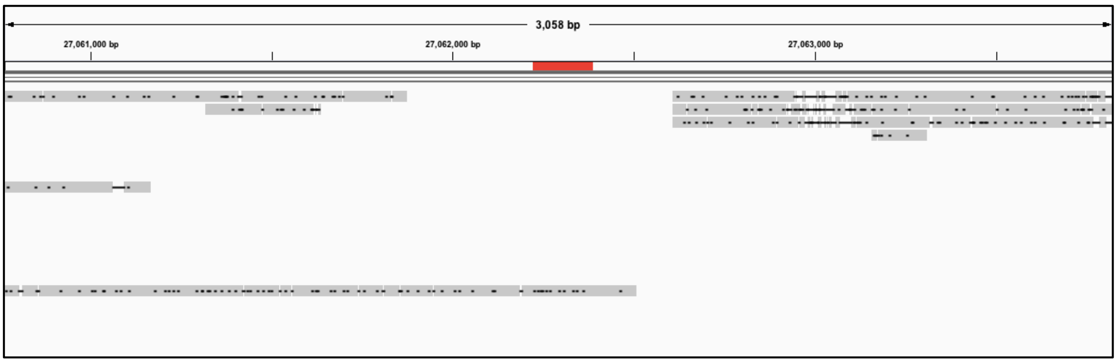

Supplementary Figure 1. PacBio read alignments of a female Atlantic cod specimen towards LG14. A single long read alignment (grey) overlaps the region containing sex-associated genotypes (red) on LG14. Small indels (black dots) within the reads are a typical feature of PacBio read data. Genomic positions are in base pair (bp). The figure is directly obtained by visualizing the read alignments using the Integrative Genomic Viewer (IGV).

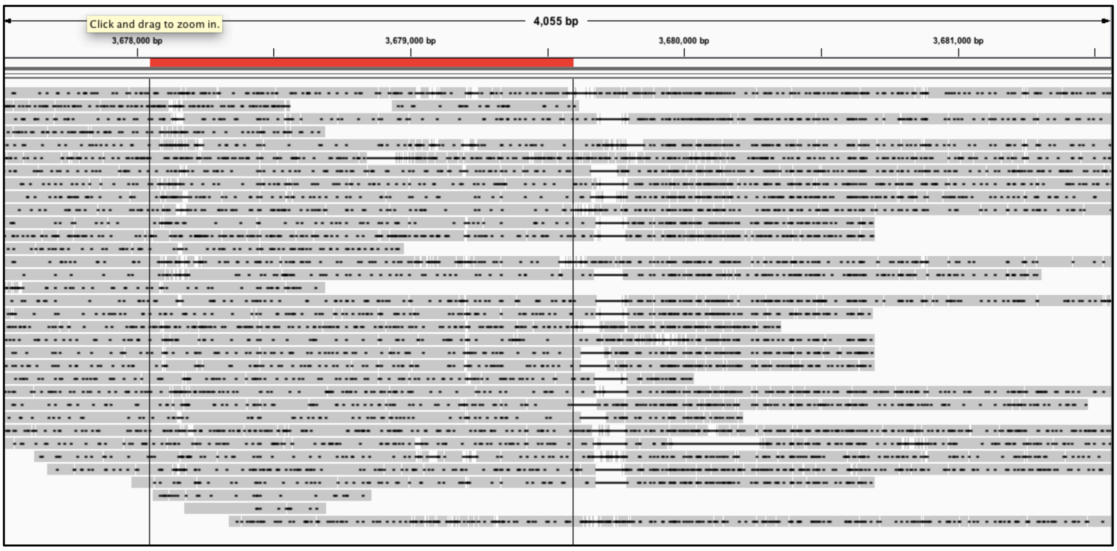

Supplementary Figure 2. PacBio read alignments of a female Atlantic cod specimen towards LG15. Long read alignments (grey) overlap the region containing sex-associated genotypes (red) on LG15. Small indels (black dots) within the reads are a typical feature of PacBio read data. Genomic positions are in base pair (bp). The figure is directly obtained by visualizing the read alignments using the Integrative Genomic Viewer (IGV).

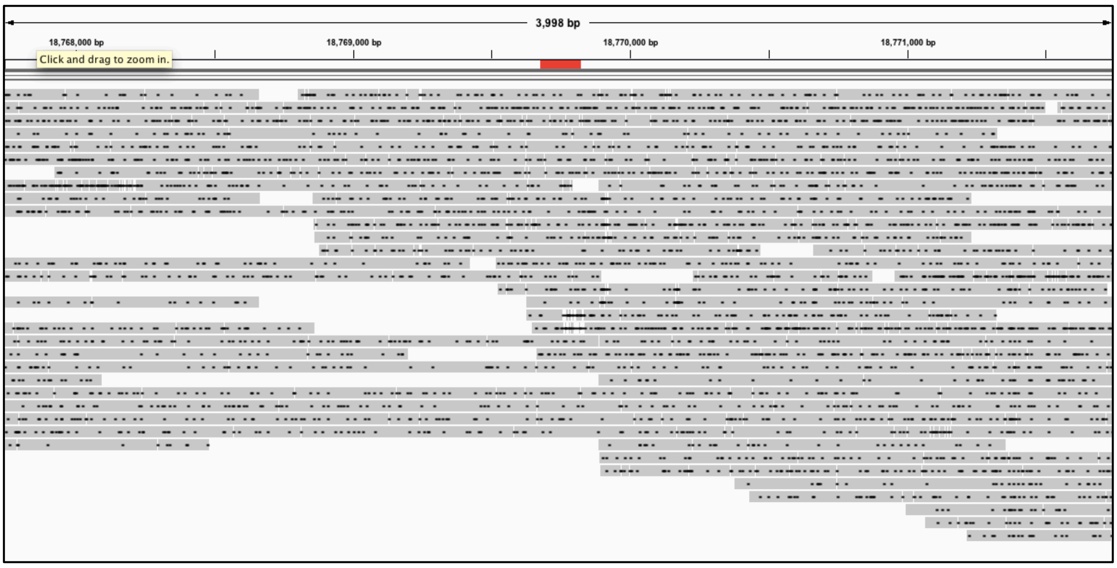

Supplementary Figure 3. PacBio read alignments of a female Atlantic cod specimen towards LG17. Long read alignments (grey) overlap the region containing sex-associated genotypes (red) on LG17. Small indels (black dots) within the reads are a typical feature of PacBio read data. Genomic positions are in base pair (bp). The figure is directly obtained by visualizing the read alignments using the Integrative Genomic Viewer (IGV).

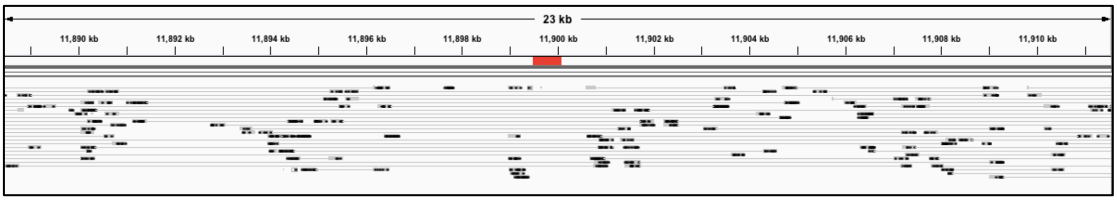

Supplementary Figure 4. Paired-end PacBio read alignments of a female Atlantic cod specimen towards LG11. Based on the full-length PacBio data of the female specimen, artificial paired-end reads were created with a length of 300 bp. The artificially created paired-end read alignments (grey lines) overlap the region (red) where their full-length equivalents fail to cross. Small indels (black dots) within the read ends are a typical feature of PacBio read data. Genomic positions are in kilobase pair (kb). The figure is directly obtained by visualizing the read alignments using the Integrative Genomic Viewer (IGV) and selecting the option “view as pairs”.

Supplementary Table 1. Location, sample name and sequencing depth for 179 Atlantic cod specimens. We predict sex based on measures of heterozygocity (F) of 13 polymorphic loci that are associated with gender in 48 Lofoten individuals. The number of homozygote (hom) and heterozygote (het) calls is shown. A single individual (AveroyaMarch1405) is misclassified based on the heterozygocity of these 13 loci.

Location	Sample name	Sequencing depth	Hom	Het	Recorded Sex	Predicted Sex	Prediction correct?
Iceland	I50_02	9.9	2	11	M	M	Y
Iceland	I50_03	10.1	2	11	M	M	Y
Iceland	I50_04	9.6	2	11	M	M	Y
Iceland	I50_05	9.9	11	0	F	F	Y
Iceland	I50_06	9.1	9	2	F	F	Y
Iceland	I50_07	10.8	2	11	M	M	Y
Iceland	I50_08	10.8	3	10	M	M	Y
Iceland	I50_09	10.7	2	11	M	M	Y
Iceland	I50_10	9.1	2	11	M	M	Y
Iceland	I50_11	11.4	2	11	M	M	Y
Iceland	I50_12	18.5	2	10	M	M	Y
Iceland	I50_13	10.2	2	11	M	M	Y
Iceland	I50_14	9.7	11	0	F	F	Y
Iceland	I50_15	12.3	11	0	F	F	Y
Iceland	I50_16	10.8	4	9	M	M	Y
Iceland	I50_17	8.4	10	0	F	F	Y
Iceland	I50_18	9.4	11	0	F	F	Y
Iceland	I50_19	9.5	11	0	F	F	Y
Iceland	I50_20	5.1	2	11	M	M	Y
Iceland	I50_23	9.2	11	0	F	F	Y
Iceland	I50_41	9.9	11	0	F	F	Y
Iceland	I50_42	12.0	11	0	F	F	Y
Averoya	AVE_M_14_01	10.6	0	13	M	M	Y
Averoya	AVE_M_14_02	8.2	13	0	F	F	Y
Averoya	AVE_M_14_05	8.8	0	13	F	M	N
Averoya	AVE_M_14_06	9.8	13	0	F	F	Y
Averoya	AVE_M_14_07	8.6	0	13	M	M	Y
Averoya	AVE_M_14_09	9.6	11	0	F	F	Y
Averoya	AVE_M_14_10	11.0	4	9	M	M	Y
Averoya	AVE_M_14_13	12.7	0	13	M	M	Y
Averoya	AVE_M_14_16	10.6	0	13	M	M	Y
Averoya	AVE_M_14_17	10.3	13	0	F	F	Y
Averoya	AVE_M_14_18	8.9	13	0	F	F	Y
Averoya	AVE_M_14_19	10.1	0	13	M	M	Y
Averoya	AVE_M_14_20	9.3	13	0	F	F	Y
Averoya	AVE_M_14_21	9.8	11	0	F	F	Y
Averoya	AVE_M_14_22	10.6	2	11	M	M	Y
Averoya	AVE_M_14_24	10.2	13	0	F	F	Y
Averoya	AVE_M_14_25	10.9	13	0	F	F	Y
Averoya	AVE_M_14_26	23.3	2	11	M	M	Y
Averoya	AVE_M_14_30	8.4	1	12	M	M	Y
Averoya	AVE_M_14_31	9.5	2	11	M	M	Y
Averoya	AVE_M_14_32	43.0	5	8	M	M	Y
Averoya	AVE_M_14_33	10.4	0	13	M	M	Y
Averoya	AVE_M_14_35	10.6	0	13	M	M	Y
Averoya	AVE_M_14_37	11.6	2	11	M	M	Y
Averoya	AVE_M_14_38	10.5	3	10	M	M	Y
Averoya	AVE_M_14_39	12.8	13	0	F	F	Y
Averoya	AVE_M_14_40	8.8	13	0	F	F	Y
Averoya	AVE_M_14_41	9.4	0	13	M	M	Y
Averoya	AVE_M_14_42	9.1	10	3	F	F	Y
Averoya	AVE_M_14_43	11.6	0	13	M	M	Y
Averoya	AVE_M_14_44	9.7	11	0	F	F	Y
Averoya	AVE_M_14_45	13.3	2	11	M	M	Y
Averoya	AVE_M_14_46	11.2	13	0	F	F	Y
Averoya	AVE_M_14_47	12.6	13	0	F	F	Y
Averoya	AVE_M_14_48	9.1	13	0	F	F	Y
Averoya	AVE_S_14_01	9.9	0	13	M	M	Y
Averoya	AVE_S_14_02	9.7	13	0	F	F	Y
Averoya	AVE_S_14_05	11.2	0	13	M	M	Y
Averoya	AVE_S_14_08	7.8	13	0	F	F	Y
Averoya	AVE_S_14_11	8.5	13	0	F	F	Y
Averoya	AVE_S_14_14	8.1	4	9	M	M	Y

Averoya	AVE_S_14_17	8.4	3	10	M	M	Y
Averoya	AVE_S_14_19	9.7	0	13	M	M	Y
Averoya	AVE_S_14_21	9.5	11	0	F	F	Y
Averoya	AVE_S_14_22	4.9	3	10	M	M	Y
Averoya	AVE_S_14_23	9.6	0	13	M	M	Y
Averoya	AVE_S_14_24	8.4	13	0	F	F	Y
Averoya	AVE_S_14_25	9.7	11	0	F	F	Y
Averoya	AVE_S_14_27	11.8	0	12	M	M	Y
Averoya	AVE_S_14_30	8.9	13	0	F	F	Y
Averoya	AVE_S_14_32	9.4	1	12	M	M	Y
Averoya	AVE_S_14_33	8.9	0	13	M	M	Y
Averoya	AVE_S_14_34	9.5	1	12	M	M	Y
Averoya	AVE_S_14_35	9.4	13	0	F	F	Y
Averoya	AVE_S_14_37	9.2	0	13	M	M	Y
Averoya	AVE_S_14_38	7.8	11	0	F	F	Y
Averoya	AVE_S_14_39	9.2	0	13	M	M	Y
Averoya	AVE_S_14_41	8.7	1	12	M	M	Y
Averoya	AVE_S_14_43	12.2	13	0	F	F	Y
Lofoten	LOF_Y_11_007	7.9	10	3	F	.	Na
Lofoten	LOF_Y_11_009	9.7	13	0	F	.	Na
Lofoten	LOF_Y_11_013	9.7	0	13	M	.	Na
Lofoten	LOF_Y_11_015	9.0	0	13	M	.	Na
Lofoten	LOF_Y_11_017	10.3	0	11	M	.	Na
Lofoten	LOF_Y_11_019	8.3	13	0	F	.	Na
Lofoten	LOF_Y_11_021	9.9	13	0	F	.	Na
Lofoten	LOF_Y_11_029	10.4	1	12	M	.	Na
Lofoten	LOF_Y_11_039	9.2	13	0	F	.	Na
Lofoten	LOF_Y_11_041	10.4	1	12	M	.	Na
Lofoten	LOF_Y_11_043	8.8	13	0	F	.	Na
Lofoten	LOF_Y_11_045	7.9	12	1	F	.	Na
Lofoten	LOF_Y_11_049	8.9	0	13	M	.	Na
Lofoten	LOF_Y_11_141	9.8	13	0	F	.	Na
Lofoten	LOF_Y_11_143	10.0	13	0	F	.	Na
Lofoten	LOF_Y_11_145	8.7	1	12	M	.	Na
Lofoten	LOF_Y_11_147	9.8	12	0	F	.	Na
Lofoten	LOF_Y_11_149	9.0	13	0	F	.	Na
Lofoten	LOF_Y_11_151	9.0	12	0	F	.	Na
Lofoten	LOF_Y_11_153	7.3	13	0	F	.	Na
Lofoten	LOF_Y_11_155	10.5	13	0	F	.	Na
Lofoten	LOF_Y_11_159	7.8	13	0	F	.	Na
Lofoten	LOF_Y_11_161	11.2	13	0	F	.	Na
Lofoten	LOF_Y_11_163	9.8	0	13	M	.	Na
Lofoten	LOF_I_11_01	9.5	13	0	F	.	Na
Lofoten	LOF_I_11_03	8.8	1	12	M	.	Na
Lofoten	LOF_I_11_04	12.4	0	13	M	.	Na
Lofoten	LOF_I_11_05	11.5	11	2	F	.	Na
Lofoten	LOF_I_11_07	10.3	0	13	M	.	Na
Lofoten	LOF_I_11_08	10.1	13	0	F	.	Na
Lofoten	LOF_I_11_09	10.4	13	0	F	.	Na
Lofoten	LOF_I_11_10	12.0	13	0	F	.	Na
Lofoten	LOF_I_11_11	11.3	0	13	M	.	Na
Lofoten	LOF_I_11_12	13.9	0	11	M	.	Na
Lofoten	LOF_I_11_13	11.7	13	0	F	.	Na
Lofoten	LOF_I_11_14	9.7	13	0	F	.	Na
Lofoten	LOF_I_11_15	8.6	13	0	F	.	Na
Lofoten	LOF_I_11_16	10.1	13	0	F	.	Na
Lofoten	LOF_I_11_17	9.9	1	12	M	.	Na
Lofoten	LOF_I_11_18	9.8	0	13	M	.	Na
Lofoten	LOF_I_11_21	11.6	1	12	M	.	Na
Lofoten	LOF_I_11_22	9.5	12	1	F	.	Na
Lofoten	LOF_I_11_23	10.0	0	13	M	.	Na
Lofoten	LOF_I_11_24	8.8	13	0	F	.	Na
Lofoten	LOF_I_11_29	12.3	0	13	M	.	Na
Lofoten	LOF_I_11_34	10.8	1	12	M	.	Na
Lofoten	LOF_I_11_35	10.3	0	13	M	.	Na
Lofoten	LOF_I_11_36	10.1	0	13	M	.	Na
Lofoten	LOF_A_14_01	11.4	2	11	M	M	Y
Lofoten	LOF_A_14_03	9.9	11	0	F	F	Y
Lofoten	LOF_A_14_04	9.7	2	11	F	F	Y
Lofoten	LOF_A_14_05	39.7	12	1	F	F	Y
Lofoten	LOF_A_14_06	10.2	0	13	F	F	Y
Lofoten	LOF_A_14_08	9.7	13	0	F	F	Y
Lofoten	LOF_A_14_09	10.3	13	0	F	F	Y
Lofoten	LOF_A_14_10	10.7	3	10	F	F	Y
Lofoten	LOF_A_14_11	10.0	0	13	M	M	Y

Lofoten	LOF_A_14_16	10.1	2	11	F	F	Y
Lofoten	LOF_A_14_17	11.3	13	0	F	F	Y
Lofoten	LOF_A_14_18	12.4	2	11	M	M	Y
Lofoten	LOF_A_14_19	12.5	13	0	F	F	Y
Lofoten	LOF_A_14_20	9.8	11	0	M	M	Y
Lofoten	LOF_A_14_21	8.9	0	13	M	M	Y
Lofoten	LOF_A_14_22	9.5	11	0	M	M	Y
Lofoten	LOF_A_14_23	9.9	11	0	F	F	Y
Lofoten	LOF_A_14_24	10.6	11	0	F	F	Y
Lofoten	LOF_A_14_25	10.6	11	0	M	M	Y
Lofoten	LOF_A_14_26	12.8	11	0	F	F	Y
Lofoten	LOF_A_14_27	11.5	11	0	M	M	Y
Lofoten	LOF_A_14_28	10.5	13	0	F	F	Y
Lofoten	LOF_A_14_29	10.0	2	11	M	M	Y
Lofoten	LOF_A_14_30	11.1	5	8	M	M	Y
Lofoten	LOF_A_14_33	10.2	0	13	M	M	Y
Lofoten	LOF_A_14_41	10.5	0	13	M	M	Y
Lofoten	LOF_M_14_26	11.6	11	0	F	F	Y
Lofoten	LOF_M_14_27	13.6	13	0	F	F	Y
Lofoten	LOF_M_14_28	12.5	0	13	M	M	Y
Lofoten	LOF_M_14_29	11.2	2	11	M	M	Y
Lofoten	LOF_M_14_30	11.1	11	0	F	F	Y
Lofoten	LOF_M_14_31	11.8	0	13	M	M	Y
Lofoten	LOF_M_14_32	8.1	0	13	M	M	Y
Lofoten	LOF_M_14_33	10.8	13	0	F	F	Y
Lofoten	LOF_M_14_35	8.7	11	0	F	F	Y
Lofoten	LOF_M_14_36	12.8	11	0	F	F	Y
Lofoten	LOF_M_14_43	11.1	0	13	M	M	Y
Lofoten	LOF_M_14_44	12.8	11	0	F	F	Y
Lofoten	LOF_M_14_45	10.1	0	13	M	M	Y
Lofoten	LOF_M_14_46	12.5	13	0	F	F	Y
Lofoten	LOF_M_14_47	6.6	13	0	F	F	Y
Lofoten	LOF_M_14_50	10.8	11	0	F	F	Y
Lofoten	LOF_M_14_51	12.7	13	0	F	F	Y
Lofoten	LOF_M_14_52	11.1	13	0	F	F	Y
Lofoten	LOF_M_14_53	11.6	13	0	F	F	Y
Lofoten	LOF_M_14_54	13.1	2	11	M	M	Y
Lofoten	LOF_M_14_55	13.7	0	13	M	M	Y
Lofoten	LOF_M_14_56	15.3	0	13	M	M	Y
Lofoten	LOF_M_14_62	10.6	0	13	M	M	Y
Lofoten	LOF_M_14_68	13.7	0	13	M	M	Y
Sorøya	SOR_M_14_06	14.8	0	13	M	M	Y
Sorøya	SOR_M_14_07	11.7	2	11	M	M	Y
Sorøya	SOR_M_14_09	9.1	11	0	F	F	Y
Sorøya	SOR_M_14_10	9.3	0	13	M	M	Y
Sorøya	SOR_M_14_11	9.6	1	12	M	M	Y
Sorøya	SOR_M_14_12	10.0	2	11	M	M	Y
Sorøya	SOR_M_14_14	12.2	0	13	M	M	Y
Sorøya	SOR_M_14_15	11.6	3	10	M	M	Y
Sorøya	SOR_M_14_16	11.6	13	0	F	F	Y
Sorøya	SOR_M_14_17	10.4	0	13	M	M	Y
Sorøya	SOR_M_14_19	10.2	11	0	F	F	Y
Sorøya	SOR_M_14_38	10.0	11	0	F	F	Y
Sorøya	SOR_M_14_39	11.3	11	0	F	F	Y
Sorøya	SOR_M_14_40	10.5	11	0	F	F	Y
Sorøya	SOR_M_14_41	12.8	11	0	F	F	Y
Sorøya	SOR_M_14_42	11.8	0	13	M	M	Y
Sorøya	SOR_M_14_43	11.5	13	0	F	F	Y
Sorøya	SOR_M_14_44	20.7	10	1	F	F	Y
Sorøya	SOR_M_14_45	11.8	11	0	F	F	Y
Sorøya	SOR_M_14_46	11.8	13	0	F	F	Y
Sorøya	SOR_M_14_47	11.1	4	9	M	M	Y
Sorøya	SOR_M_14_48	14.0	13	0	F	F	Y
Sorøya	SOR_M_14_49	11.3	2	11	M	M	Y
Sorøya	SOR_M_14_50	13.0	11	0	F	F	Y
Sorøya	SOR_S_13_39	9.8	12	0	F	F	Y
Sorøya	SOR_S_13_40	10.6	2	11	M	M	Y
Sorøya	SOR_S_13_42	10.9	2	11	M	M	Y
Sorøya	SOR_S_13_45	14.7	13	0	F	F	Y
Sorøya	SOR_S_13_46	11.4	0	13	M	M	Y
Sorøya	SOR_S_13_49	13.3	2	11	M	M	Y
Sorøya	SOR_S_13_50	11.0	0	13	M	M	Y
Sorøya	SOR_S_13_52	10.8	13	0	F	F	Y
Sorøya	SOR_S_13_53	10.7	11	0	F	F	Y
Sorøya	SOR_S_13_54	11.5	11	0	F	F	Y

Sorøya	SOR_S_13_55	10.0	13	0	F	F	Y
Sorøya	SOR_S_13_56	12.1	11	0	F	F	Y
Sorøya	SOR_S_13_59	11.8	2	11	M	M	Y
Sorøya	SOR_S_13_64	14.0	13	0	F	F	Y
Sorøya	SOR_S_13_66	12.0	13	0	F	F	Y
Sorøya	SOR_S_13_67	11.8	0	13	M	M	Y
Sorøya	SOR_S_13_69	11.1	0	13	M	M	Y
Sorøya	SOR_S_13_71	11.2	0	13	M	M	Y
Sorøya	SOR_S_13_72	9.7	13	0	F	F	Y
Sorøya	SOR_S_13_76	11.7	0	13	M	M	Y
Sorøya	SOR_S_13_78	10.7	11	0	F	F	Y
Sorøya	SOR_S_13_79	10.5	0	13	M	M	Y
Sorøya	SOR_S_13_81	12.0	13	0	F	F	Y
Sorøya	SOR_S_13_85	13.4	13	0	F	F	Y

Supplementary Table 2. Position, allelic and genotypic composition of sex-linked loci with *p*-values at least six Standard Deviation (SD) away from the mean. *P*-values were calculated comparing genotypes of Atlantic cod females and males using Fisher's exact test. Values are –log transformed and loci are sorted according to *p*-value.

LG	Position	Allele Reference	Alternative	Genotype (*ref.het.alt*) Females	Males	-log(*p*)
LG11	11888434	T	C	114.0.0	2.109.1	66.7228
LG11	11897471	G	GTGT	0.0.114	2.107.3	64.6546
LG11	11897519	A	T	0.0.114	2.107.3	64.6546
LG11	11895561	G	C	0.0.113	7.102.3	64.3687
LG11	11897588	T	G	0.0.113	5.102.3	63.745
LG11	11897566	AATCC	A	0.1.111	2.106.2	63.4844
LG11	11897513	C	T	0.0.114	2.106.4	62.8837
LG11	11886873	T	A	0.1.113	3.106.3	62.6169
LG11	11885753	G	T	0.2.110	4.105.2	62.0163
LG11	11899391	C	CT	0.2.112	0.109.3	60.8761
LG11	11899196	G	T	0.1.113	0.107.5	59.2628
LG11	11897286	CAA	C	0.1.111	29.70.3	58.9017
LG11	11899495	T	C	0.0.87	38.71.2	57.9197
LG11	11899188	A	G	0.1.113	0.106.6	57.7934
LG11	11899373	T	A	0.1.113	0.105.7	56.4171
LG11	11884677	T	TTTCGGGGTC	0.4.110	9.98.2	55.4612
LG11	11899487	C	A	0.1.84	38.71.2	55.1811
LG11	11884313	G	A	0.6.108	4.105.3	54.759
LG11	11893931	G	A	0.0.113	4.98.10	54.4203
LG11	11893943	A	G	0.0.113	3.99.10	54.4203
LG11	11893118	T	A	114.0.0	10.99.2	54.3683
LG11	11899288	A	C	0.0.114	0.101.11	53.5384
LG11	11899355	C	A	0.0.114	0.101.11	53.5384
LG11	11899434	T	TTA	0.1.81	28.81.1	53.4522
LG11	11899440	C	A	0.1.81	26.80.1	53.0224
LG11	11899305	C	G	114.0.0	12.100.0	52.4379
LG11	11899306	G	A	114.0.0	12.100.0	52.4379
LG11	11900583	T	G	2.1.97	43.64.5	52.0255
LG11	11899287	C	G	0.1.113	0.101.11	51.5618
LG11	11890528	T	TA	114.0.0	13.98.1	51.3755
LG11	11890530	C	G	114.0.0	13.98.1	51.3755
LG11	11890531	T	A	114.0.0	13.98.1	51.3755
LG11	11892971	A	G	114.0.0	13.98.1	51.3755
LG11	11899309	T	C	114.0.0	13.99.0	51.3755
LG11	11900627	T	A	3.2.100	37.70.5	51.022
LG11	11899344	A	C	114.0.0	14.98.0	50.3476
LG11	11887720	C	T	0.2.112	5.95.11	49.4487
LG08	1784612	G	C	114.0.0	17.95.0	47.4419
LG11	11899539	G	T	0.3.69	37.72.1	46.9034
LG11	11897239	C	T	0.0.112	25.63.15	46.0074
LG11	11899548	TA	T	0.3.69	37.71.2	45.059
LG11	11899455	C	G	0.1.61	62.39.1	44.3422
LG11	11899456	C	T	0.1.61	62.39.1	44.3422
LG11	11892844	G	A	114.0.0	21.84.6	43.5959
LG08	1784766	A	C	114.0.0	24.88.0	41.4642
LG11	11900496	A	C	2.1.55	81.29.1	40.9179
LG15	3678047	G	A	114.0.0	25.87.0	40.6828
LG11	11899462	A	G	0.1.52	68.32.1	40.6381
LG11	11897211	G	A	0.1.110	20.63.19	39.9097
LG11	11899589	A	G	1.4.58	56.48.2	38.2237
LG11	11899590	T	C	1.4.58	56.48.2	38.2237
LG11	11897256	G	T	106.0.0	19.65.10	38.0784

LG11	11882114	G	A	1.12.101	8.91.12	35.8416
LG11	11882397	A	G	1.11.102	11.87.14	34.8153
LG11	11868892	A	G	0.7.107	8.82.22	33.0032
LG11	11882972	A	G	100.14.0	15.92.4	32.2323
LG11	11866841	T	A	108.6.0	26.85.1	31.5318
LG11	11881590	CAAA	C	102.12.0	18.89.5	31.5233
LG11	11878389	C	A	101.13.0	17.91.4	31.4947
LG11	11878390	A	T	101.13.0	17.91.4	31.4947
LG11	11882191	G	C	1.12.101	6.89.17	31.4544
LG11	11879920	C	G	106.8.0	24.78.10	30.5634
LG15	3678509	C	T	113.1.0	38.72.2	29.9292
LG11	11897888	C	T	0.0.29	82.27.0	29.734
LG11	11893394	G	T	113.0.0	32.52.6	28.0626
LG11	11896849	A	T	111.0.0	30.49.8	28.053
LG11	11871538	G	A	0.14.100	10.81.21	27.6979
LG11	11897183	AT	A	113.0.0	35.51.5	26.4359
LG11	11897094	T	G	0.0.45	55.10.6	26.3407
LG17	18769711	G	A	113.1.0	45.67.0	25.8671
LG08	1784400	A	G	114.0.0	50.62.0	24.8303
LG08	1784404	A	G	114.0.0	50.62.0	24.8303
LG08	1784429	ACGAT	A	109.0.0	51.60.0	23.3098
LG08	1784813	G	T	114.0.0	53.59.0	23.2669
LG11	11897743	GGTACTAAA	G	0.0.20	84.24.0	23.0779
LG11	11878698	A	G	105.7.0	35.62.10	22.7238
LG11	11897815	AATC	A	4.0.26	82.26.2	22.6057
LG11	11897935	G	C	0.0.19	84.25.0	22.3415
LG14	27062326	G	A	114.0.0	54.56.0	22.1301
LG15	3678607	A	G	114.0.0	56.54.1	21.4432
LG11	11879620	A	G	2.9.99	27.42.26	21.4195
LG11	11897771	ATAAATG	A	0.1.20	84.23.1	21.163
LG11	11877918	G	C	100.8.2	27.48.19	21.0144
LG11	11870831	T	C	109.5.0	45.66.1	20.9439
LG08	1784804	G	A	113.1.0	54.58.0	20.9355
LG11	11902824	TTTTC	T	0.36.78	11.88.13	20.7918
LG11	11897795	ATAAAAGTG	A	1.0.26	39.25.2	20.4024
LG11	11864114	A	G	0.9.105	5.68.39	20.309
LG17	18769822	G	A	114.0.0	59.53.0	20.2937
LG11	11895278	A	G	112.0.0	38.38.3	19.9187
LG11	11897835	A	G	6.2.26	82.24.2	19.7014
LG11	11887941	A	T	19.35.57	54.55.2	19.6578
LG11	11896785	T	G	104.0.0	37.32.10	19.6572
LG11	11864338	A	C	106.8.0	42.67.2	19.4949
LG14	27062388	C	A	114.0.0	61.51.0	19.3444
LG08	24118823	A	AG	27.0.0	6.19.54	19.0263
LG11	11898436	C	G	11.53.50	34.0.77	18.766
LG15	3678560	T	G	113.0.0	58.47.1	18.7615
LG11	11884987	A	C	14.48.52	45.66.1	17.4012
LG15	3679388	G	T	88.0.0	41.31.12	17.2651
LG11	11885065	T	A	17.44.53	47.64.1	17.2547
LG17	18769676	G	A	114.0.0	66.46.0	17.0538
LG11	11898609	A	G	59.47.8	78.0.31	16.9876
LG11	11897878	T	TGC	8.1.20	86.23.0	16.5674
LG11	11898425	C	T	62.45.7	71.2.38	16.1478
LG08	1784756	T	C	67.47.0	12.100.0	15.6388
LG11	11897905	C	A	7.1.18	89.21.0	15.4019
LG11	11904163	T	G	0.1.110	6.39.66	14.6938
LG15	3679415	G	A	91.0.0	50.21.17	14.479
LG08	24118567	G	A	59.0.0	30.22.22	14.3576
LG11	11886135	C	T	41.57.16	1.58.53	14.1749
LG08	24118576	G	T	58.0.0	30.22.21	13.9087

LG15	3679605	C	A	80.0.0	36.12.19	12.9851
LG11	11897873	ACC	A	23.1.20	89.23.0	12.8292
LG11	11898639	A	G	1.30.82	17.0.87	12.1666
LG11	11907860	A	G	0.2.112	3.38.70	12.0979
LG08	24118529	A	C	47.0.0	24.12.23	12.0024
LG11	11880247	C	G	111.3.0	69.41.2	11.8719
LG11	11894953	TGCAGGCAGGG	T	81.23.4	32.46.29	11.8586
LG15	3679348	A	C	72.1.0	39.17.18	11.6158
LG08	24118864	T	TAA	27.0.1	13.12.38	11.3835
LG11	11904283	T	C	0.3.104	12.27.59	11.3144
LG08	1784577	C	A	0.70.44	2.104.5	11.0058
LG14	27062221	C	T	99.15.0	51.61.0	10.9763
LG15	3679330	T	C	68.0.0	39.11.18	10.6313
LG11	11869268	C	A	110.4.0	71.40.1	10.2406
LG11	11898204	CTGCATT	C	87.26.1	92.2.16	9.78888
LG15	3679601	A	G	7.4.45	44.17.19	9.40627
LG15	3678656	A	T	69.43.0	22.78.7	9.37882
LG11	11883036	G	A	36.52.26	2.61.49	9.09066
LG11	11888226	A	G	25.55.34	0.47.65	8.91614
LG11	11882287	G	T	36.58.20	5.56.51	8.62599
LG15	3679328	G	A	62.1.2	32.13.19	8.57741
LG11	11909716	A	G	3.31.80	13.65.34	8.48892
LG11	11884966	A	G	33.58.23	64.48.0	8.47224
LG15	3679314	T	A	63.1.2	34.14.18	8.36562
LG11	11888804	C	T	38.48.28	69.42.1	8.35301
LG11	11897707	A	G	0.0.5	82.27.0	8.16673
LG11	11884428	G	T	34.57.23	63.49.0	8.09162
LG11	11887651	C	T	35.57.22	65.47.0	7.9914
LG08	24118526	T	G	26.0.1	15.10.26	7.94885
LG08	1784484	G	T	10.76.28	8.100.2	7.93517
LG08	1784427	A	G	36.44.0	10.95.1	7.89143
LG11	11889859	A	C	27.54.33	2.45.65	7.58403
LG08	24118524	C	A	26.0.1	16.10.26	7.54607
LG08	24118777	T	G	19.3.4	9.12.45	7.06808
LG11	11910235	T	A	5.37.72	13.69.30	7.00358
LG15	3679420	T	C	49.11.13	17.32.34	6.99268
LG11	11884616	G	T	35.56.23	52.59.0	6.91578
LG11	11918378	A	T	104.10.0	69.40.2	6.89757

Supplementary Table 3. Sample location, date and number of Atlantic cod (*Gadus morhua*) specimens (♂ and ♀) used in this study.

Sample Location	Year	No. of specimens	Males	Females
Iceland	2003	22	12	10
Lofoten, Norway	2011	48	21	27
Lofoten, Norway	2014	50	23	27
Averøya, Norway	2014	59	32	27
Sorøya, Norway	2014	48	22	26
Total		227	110	117
